# An Elusive and Deceptive Tachycardia

**DOI:** 10.19102/icrm.2017.080202

**Published:** 2017-02-15

**Authors:** Sujata Balulad, Subbarao Choudry, William Whang, Vivek Y. Reddy

**Affiliations:** ^1^Icahn School of Medicine at Mount Sinai, New York, NY

**Keywords:** Arrhythmias, catheter ablation, wide-complex tachycardia

## Abstract

A wide-complex tachycardia was induced with rapid atrial pacing, with intermittent ventriculoatrial block and QRS alternans. A short/negative HV interval was observed, and premature ventricular complexes resulted in the termination of the tachycardia. The findings at electrophysiology study were consistent with an antidromic re-entrant tachycardia involving a nodo-fascicular/ventricular connection.

## Case presentation

**Figure 1:**
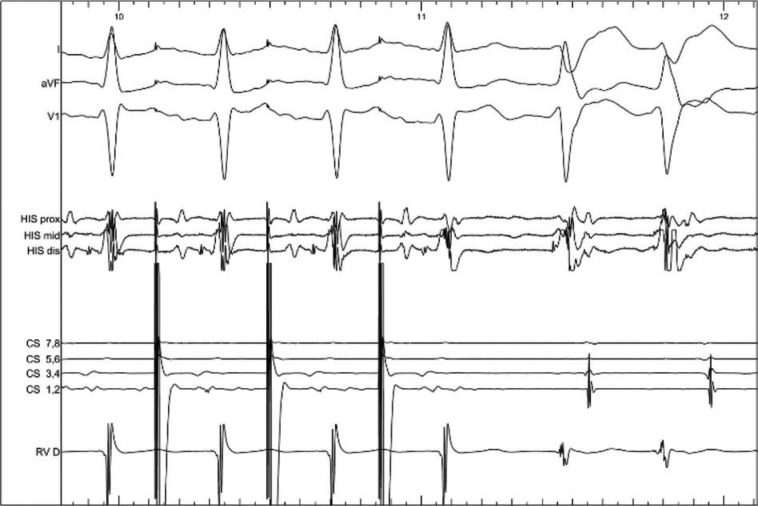
Initiation of sustained tachycardia with rapid atrial pacing.

A 30-year-old woman with no known structural heart disease and repeated episodes of narrow complex tachycardia by Holter monitor presented for electrophysiology study and catheter ablation. Antegrade conduction was midline and decremental, and there was no evidence of pre-excitation at baseline. Retrograde conduction with right ventricular pacing was absent at baseline and with isoproterenol infusion. Tachycardia was difficult to initiate, but was ultimately induced with burst atrial pacing after administration of isoproterenol and atropine **(Figure 1)**. What was the mechanism of the tachycardia?

## Discussion

**Figure 2:**
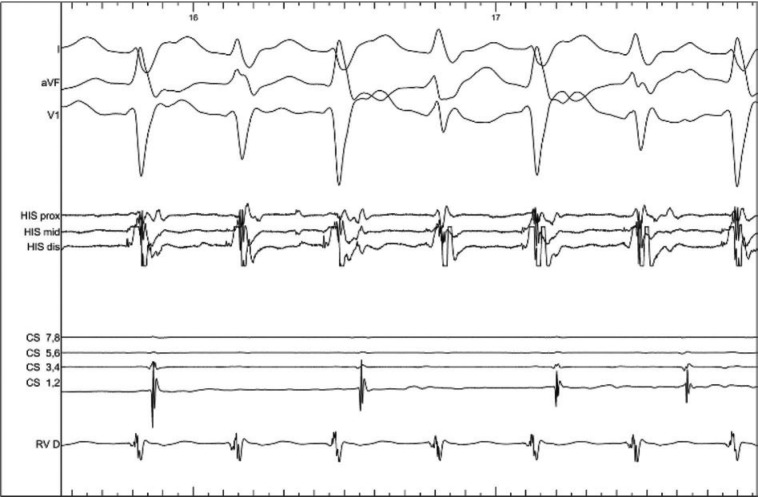
During tachycardia, ventriculoatrial dissociation is present as indicated in the catheter labeled “CS,” which was repositioned from the coronary sinus to the lateral right atrium during this part of the case. Alternating QRS morphology is present.

Our patient’s tachycardia had a left bundle branch block/intraventricular conduction delay pattern, QRS duration of 104 ms, and short or negative HV interval. Ventriculoatrial dissociation was observed during tachycardia, ruling out an atrioventricular connection, and QRS alternans was also noted **(Figure 2)**. The differential diagnosis of the tachycardia included antidromic re-entrant tachycardia involving a nodo-fascicular/ventricular pathway, idiopathic ventricular tachycardia with intermittent alternating exits, and, much less likely, AV node re-entry or junctional tachycardia with a bystander nodo-fascicular connection and intermittent block in the upper common pathway.

Unfortunately, the tachycardia was not sustained long enough to pursue maneuvers such as entrainment with atrial pacing in order to differentiate supraventricular from ventricular tachycardia (the latter would have been expected to result in an “AVVA” response). All told, the patient’s documented narrow complex tachycardia by Holter monitor, in addition to the findings from the current study, led us to surmise that a nodo-fascicular pathway was participating in both an antidromic and orthodromic tachycardia.^1^ A radiofrequency ablation was delivered in the anatomic slow pathway, and tachycardia was not inducible afterwards.
